# 2D–2D heterostructure g-C_3_N_4_-based materials for photocatalytic H_2_ evolution: Progress and perspectives

**DOI:** 10.3389/fchem.2022.1063288

**Published:** 2022-12-12

**Authors:** Rashid Mehmood, Zia Ahmad, Muhammad Bilal Hussain, Muhammad Athar, Ghulam Akbar, Zeeshan Ajmal, Sikandar Iqbal, Rameez Razaq, Mohammad Arif Ali, Abdul Qayum, Aadil Nabi Chishti, Fakhr uz Zaman, Rahim Shah, Shahid Zaman, Adnan

**Affiliations:** ^1^ Institute of Chemical Sciences, Bahaudin Zakariya University, Multan, Pakistan; ^2^ Department of Chemistry and Biochemistry, University of Agriculture, Faisalabad, Pakistan; ^3^ School of Energy and Power Engineering, Shandong University, Jinan, China; ^4^ Department of Soil and Environmental Science, University of Agriculture, Faisalabad, Pakistan; ^5^ ZJU-Hangzhou Global Technological and Innovation Center, Zhejiang University, Hangzhou, China; ^6^ Department of Chemical Engineering, Norwegian University of Science and Technology (NTNU), Trondheim, Norway; ^7^ Institute of Chemistry, The Islamia University of Bahawalpur, Bahawalpur, Pakistan; ^8^ Department of Chemistry, Shantou University, Shantou, China; ^9^ School of Materials Science and Engineering, University of Jinan, Jinan, China; ^10^ Institute of Chemical Sciences University of Swat, Swat, Khyber Pakhtunkhwa, Pakistan; ^11^ Department of Mechanical and Energy Engineering, Southern University of Science and Technology (SUTech), Shenzhen, China

**Keywords:** photocatalytic H2 evolution, two dimensional, graphitic carbon nitride, heterojunction, sustainable energy

## Abstract

Photocatalytic hydrogen generation from direct water splitting is recognized as a progressive and renewable energy producer. The secret to understanding this phenomenon is discovering an efficient photocatalyst that preferably uses sunlight energy. Two-dimensional (2D) graphitic carbon nitride (g-C_3_N_4_)-based materials are promising for photocatalytic water splitting due to special characteristics such as appropriate band gap, visible light active, ultra-high specific surface area, and abundantly exposed active sites. However, the inadequate photocatalytic activity of pure 2D layered g-C_3_N_4_-based materials is a massive challenge due to the quick recombination between photogenerated holes and electrons. Creating 2D heterogeneous photocatalysts is a cost-effective strategy for clean and renewable hydrogen production on a larger scale. The 2D g-C_3_N_4_-based heterostructure with the combined merits of each 2D component, which facilitate the rapid charge separation through the heterojunction effect on photocatalyst, has been evidenced to be very effective in enhancing the photocatalytic performance. To further improve the photocatalytic efficiency, the development of novel 2D g-C_3_N_4_-based heterostructure photocatalysts is critical. This mini-review covers the fundamental concepts, recent advancements, and applications in photocatalytic hydrogen production. Furthermore, the challenges and perspectives on 2D g-C_3_N_4_-based heterostructure photocatalysts demonstrate the future direction toward sustainability.

## Introduction

Energy, along with environmental issues, has become increasingly important in recent decades; however, renewable energy alternatives such as wind or solar energy are essential to lessen the provoked global energy shortage (Hou et al., 2013; Qi et al., 2017; Zhu et al., 2018; Zhao et al., 2021; Ran et al., 2022). In various catalytic processes, 2D g-C_3_N_4_ layered composite materials are effective catalysts because of their visible range (2.7 eV) band gap, wavelength (∼460 nm), photo-responsive character, special geometry, and the presence of numerous N-based molecules to stabilize the metal nanoparticles. Furthermore, 2D g-C_3_N_4_ also have the ability to produce coordinative unsaturated metal centers than their 3D counterparts, which are sometimes even more active and stable than 3D or 1D materials (Yan et al., 2016; She et al., 2017; Wang et al., 2020; Tran Huu et al., 2021). Interestingly, the conduction band (CB) bottom of g-C_3_N_4_ (−1.13 eV) is more negative as compared to the water reduction potential (H_2,_ 0 V), whereas the uppermost valence band (VB) is in a slightly higher positive state than the water oxidation potential (O_2_). Hence, g-C_3_N_4_ can be utilized for water oxidation (WOR) in addition to the reduction of water (HER) (Yang et al., 2018; Wang et al., 2020). However, the g-C_3_N_4_ demonstrates constrained photocatalytic efficiency in terms of low activity as the electron and hole pairs show rapid recombination (Wang et al., 2009; Wang et al., 2014a; Wang et al., 2017; Huang et al., 2019). That is the reason that the g-C_3_N_4_ only performs photocatalytic HER to only a limited µmoles per gram per hour and is even less activated for water oxidation. Overall, nitrogen atoms in g-C_3_N_4_ are at an ideal oxidation position for water to O_2_ molecule, and the C atom serves as an active site for H^+^ ion for the generation of H_2_ (Wang et al., 2011; Ong et al., 2016). It should be noted that an effective approach to substantially enhance the photocatalytic activity of g-C_3_N_4_ material is to investigate the carbon and nitrogen atoms substitution as porous surfaces in 2-dimensional g-C_3_N_4_. Nevertheless, the challenge of rapid recombination of electron and hole pairs and excitonic characteristics continued to restrict the efficiency of the g-C_3_N_4_ photocatalysts. To further improve the photocatalytic efficiency of g-C_3_N_4_, and to overcome the problem of rapid electron and hole pairs recombination, various strategies, including structural engineering (Liu et al., 2018), surface modification (Liu et al., 2020), forming composites or heterostructure with other semiconductors (Zheng et al., 2020), doping of metals and non-metals (Wang et al., 2017), or the co-catalysts have been investigated.

**FIGURE 1:**
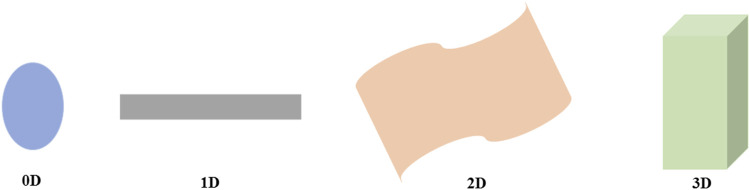
Schematic representation of photocatalysts relative to the different dimensions.

The most effective strategy is to construct the heterostructure/heterojunction involving 2D g-C_3_N_4_ and 2-dimensional material for the spatial departure of photo-generated electron and hole pairs (Fu et al., 2019; Yuan et al., 2019). The formation of heterojunction of two-dimensional designed structures along with the highly intact interface is among the key factors which usually support the electronic cloud transmission between the two materials (Ran et al., 2018b; Yang et al., 2018). In addition, the ultrathin nanosheets of 2D heterostructure produce an abundant catalytic active site, which decreases the transfer distance and improves the light absorption capacity. Moreover, the 2D heterostructure photocatalysts are exceptionally stable. That is why constructing multiple 2D materials to create 2D/2D heterostructure photocatalysts has recently gained considerable interest (Su et al., 2019; Sun et al., 2019). Moreover, photo-catalytically induced electron–hole pair clouds of g-C_3_N_4_ may be amended and deviate from the exciton severance in g-C_3_N_4_. Thus, these generated electron holes may be separated enough to improve the 2D g-C_3_N_4_ photocatalytic action. Different dimensions of photocatalysts are shown in Figure 1.

### Basic mechanism of photocatalytic water splitting

**FIGURE 2:**
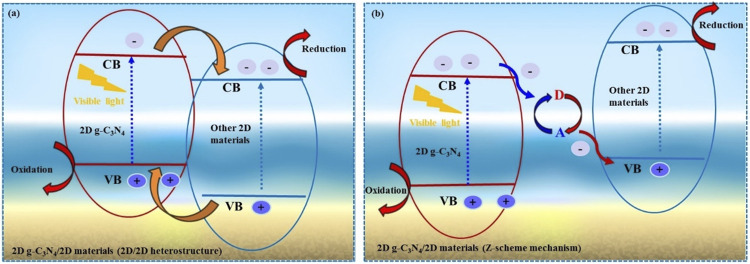
Schematic illustration of fundamental of photocatalytic water splitting for 2D g-C_3_N_4_ based 2D-2D heterostructure photocatalysts. **(A)**, type-II photocatalytic mechanism, **(B)** Z-scheme mechanism. CB (conduction band), VB (valance band); Eg, (band gap).

The semiconductor material excited by light irradiation of more intensity or band gap equivalent intensity drives the photocatalytic H_2_O splitting. During this scenario, electron flow takes place from the VB to the CB, whereas the hole (h^+^) remains in the VB of the material. The photo-generated electrons (e^−^) and holes (h^+^) potentially reduce H^+^ and oxidize the H_2_O. In this case, if the bottom of CB is more negative relative to the H^+^ ion reduction potential, it can be a suitable candidate for water reduction. As illustrated in Figure 2A, the VB value should be greater than the H_2_O molecule’s oxidation potential to generate an O_2_ molecule (1.23 eV vs. NHE at pH = 0). Furthermore, the semiconductor band gap value should be greater than the thermodynamic requirement of 1.23 eV, and it must span the redox potential of H_2_O in order to be a viable candidate for one-step excitation water splitting (Hisatomi et al., 2014). Recombining the photoinduced electronic pair clouds can happen in bulk and during a photocatalytic H_2_O response to the bulk catalyst surface. Recombination of electron and hole pairs may reduce the photocatalyst performance.

### Z-scheme

In 1979, the traditional Z-scheme photocatalyst was proposed (Bard, 1979). The components of this photocatalytic system are two photocatalysts and a redox couple. The redox couple consists of an electron acceptor (A) and an electron donor (D), including I_3_^−^/I^−1^ and Fe^+3/^Fe^+2^. In the conventional Z-scheme mechanism (Figure 2B), photogenerated electrons in the CB of 2D g-C_3_N_4_ react with A to form D, while photogenerated holes in the VB of other 2D catalysts react with D to form A. As a result, the electrons in other 2D catalysts CB and the holes in 2D g-C_3_N_4_ VB are preserved. However, traditional Z-scheme mechanisms have limitations and drawbacks. Due to the necessity of redox ion pairs, traditional Z-scheme photocatalysts are only applicable in the solution phase.

### S-scheme

The S-scheme heterojunction, which is comprised of an oxidation photocatalyst (OP) and a reduction photocatalyst, was proposed to overcome the inadequacy of traditional type-II heterojunction and Z-scheme heterojunction (RP) (Xu et al., 2020). Overall, the S-scheme mechanism vividly describes the charge transfer pathway in heterojunction photocatalysts, but it is also consistent with the scientific principles and experimental phenomena. The S-scheme heterojunction photocatalyst has both high charge separation efficiency and potent redox capability. As anticipated, it has received a great deal of attention since its proposal. Numerous sources discuss the fabrication and photocatalytic performance of 2D/2D g-C_3_N_4_-based S-scheme heterojunction photocatalysts (g-C_3_N_4_/BiOBr) (Zhang et al., 2021).

This mini-review focuses on significant and advanced phenomena in engineering 2D g-C_3_N_4_-based heterostructure photocatalysts, particularly for hydrogen production. The main aspects of 2D g-C_3_N_4_ heterogeneous photocatalysts often provide some rising strategies for contriving various 2D heterostructure photocatalysts. Further, it also provides an understanding of the designs of g-C_3_N_4_-based heterogeneous catalysts, along with special attention to the underlying mechanism of photocatalyzed recombination of electron–hole pairs. Moreover, the recent advancement and challenges of g-C_3_N_4_-based heterostructure photocatalysts for H_2_ production have subsequently been highlighted. Finally, a brief overview of 2D heterogeneous photocatalysts relevant to water reduction (H_2_ evolution) or water oxidation (O_2_ evolution) Z-scheme is described, and the current state of science and key questions are addressed.

### Structure and properties of g-C_3_N_4_

**FIGURE 3:**
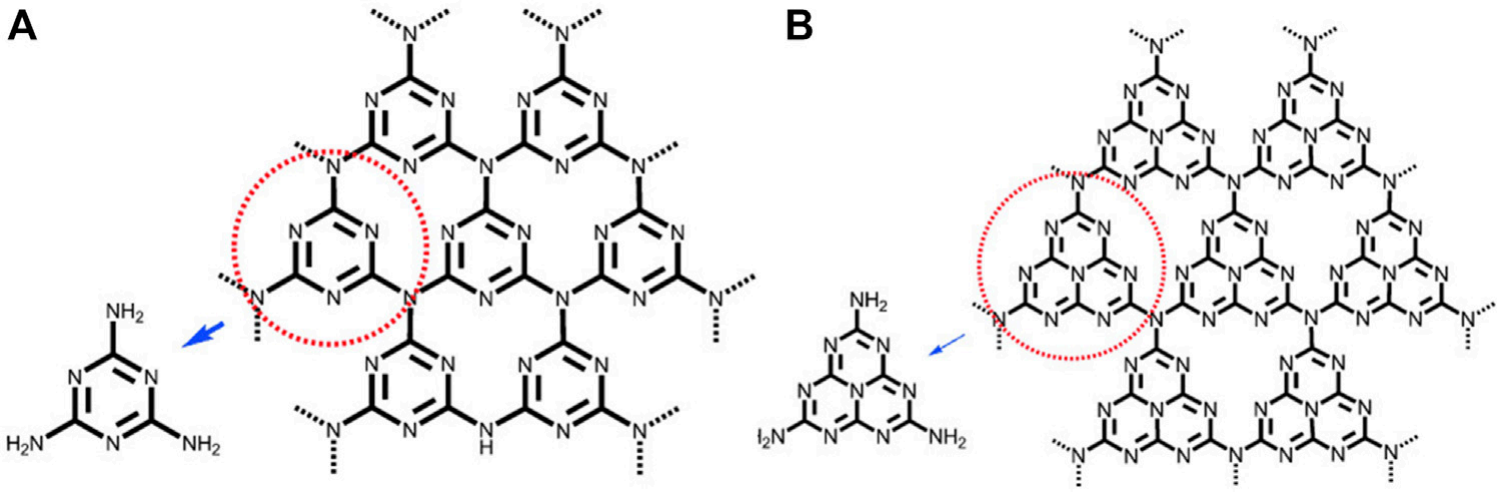
**(A)** Triazine and **(B)** tri-s-triazine (heptazine) structures of g-C_3_N_4_ (gray, blue, and white balls are carbon, nitrogen, and hydrogen, respectively) (Wang et al., 2012), Copyright 2012 Wiley Online Library.

Melamine, melon, melam, and melem are recognized as heptazine and triazine-based molecular compounds, whereas the coplanar tri-s-triazine unit is regarded as the fundamental structural motif required to produce g-C_3_N_4_. As shown in Figure 3, the basic tectonic units of g-C_3_N_4_ are triazine (C_3_N_3_) and tri-s-triazine/heptazine (C_6_N_7_) rings. Unlike conventional organic semiconductor materials, g-C_3_N_4_ cases have large stability, including resistance to heat and chemicals. Thermal gravimetric analysis (TGA) and thermal gravimetric analysis (TG) on g-C_3_N_4_ indicate that it is non-volatile up to 600°C and will be nearly completely decomposed at 700°C. The flake-like structure of g-C_3_N_4_ is very similar to that of graphite, as is well known.

### 2D g-C_3_N_4_ as photocatalysts

Wang et al., for the first time, used g-C_3_N_4_ as visible light photocatalysts for hydrogen production (Wang et al., 2009). Graphitic carbon nitride was later discovered to absorb visible light owing to its 2.7 eV band gap value, which is consistent with a wavelength of 460 nm. Moreover, the CB of g-C_3_N_4_ in its bottommost is more negative relative to the H_2_O reduction potential of the H_2_ molecule. The valence band uppermost region is more positive as compared to the oxidation potential of water to O_2_ molecule. So, graphitic carbon nitride applied as a photocatalyst could be a suitable candidate for H_2_O splitting in visible light (Yan et al., 2016). It is very imperative to understand the band distance of g-C_3_N_4_ upsurges as the bulk graphitic carbon nitride flake off to monolayer by quantum confinement effect.

## Principles of 2D g-C_3_N_4_-based 2D heterostructure photocatalysts

The basic principle to design the 2D heterostructure photocatalysts is to overwhelm the hole pair recombination issues in primeval g-C_3_N_4_. On the basis of continuous efforts, it is concluded that an appropriate heterogeneous structure is described as the utmost viable approach to increase the lifetime of electron–hole pair clouds that significantly improve the catalysts’ photocatalytic efficiency. Overall, the 2D interface design strategy is essential for 2D photocatalysts and photocatalytic performance, as the synthesis methods determine the quality of the interface in the heterostructure materials.

### Preparation of 2D g-C_3_N_4_-based heterostructure photocatalysts

The strategies to fabricate the 2D–2D g-C_3_N_4_-based hetero-structured photocatalysts Interfaces play a crucial role in the photocatalytic performance of the 2D photocatalysts, as the quality of the interface is determined by the construction strategy. To date, numerous effective fabrication techniques for the synthesis of 2D–2D g-C_3_N_4_-based heterostructure photocatalysts, such as ultrasonic absorption (Zhang et al., 2018; Ayodhya and Veerabhadram, 2020), hydrothermal method (Tian et al., 2013), electrostatic self-assembly (Ma et al., 2017; Wang et al., 2022), and chemical vapor deposition (Zhang and Fu, 2018), have been extensively studied. Nonetheless, one of the simplest ways to construct the 2D–2D g-C_3_N_4_-based heterostructure is to disperse the two distinct 2D components in the solution *via* stirring or sonication to form a mixture. After drying the mixture in an oven to evaporate the solvents, the 2D–2D photocatalyst can be obtained. Using ultrasonic dispersion and drying, 2D–2D g-C_3_N_4_/N-doped La_2_Ti_2_O_7_ layered heterostructures were fabricated. Nevertheless, these 2D–2D interfaces were successfully fabricated by a weak interaction between two 2D components, and the 2D components were easily separated during the photocatalysis process (Cai et al., 2017b). However, using a hydrothermal method, this issue can be resolved. For instance, g-C_3_N_4_/ZnIn_2_S_4_ (Manchala et al., 2019) and TiO_2_-g-C_3_N_4_ (Zhang et al., 2017) heterostructure photocatalysts, *etc.,* have been prepared by the hydrothermal method, and numerous intimate interfaces were formed between the g-C_3_N_4_ and the second counterparts. The heterointerface junctions not only enhance the stability but also enhance the generation of electron–hole pairs and inhibit their recombination. Overall, the hydrothermal method is an energy-efficient and environmentally friendly method because the reaction occurs under closed system conditions. Furthermore, the hydrothermal method is kinetically slow at all temperatures, making it easy to control. To further enhance the kinetics of crystallization, a microwave–hydrothermal method was also developed. For instance, TiO_2_/g-C_3_N_4_ heterostructures were created by a simple microwave–hydrothermal process, which demonstrated enhanced photocatalytic H_2_ production activity compared to TiO_2_ (Girish et al., 2022). Additionally, electrostatic self-assembly is a viable technique for fabricating intimate 2D–2D interfaces. Notably, surface charge modification plays a significant role in the engineering of 2D–2D photocatalysts with intimate interfacial contact using this method (Ma et al., 2017; Wang et al., 2022). To achieve electrostatic self-assembly, the surface charges on various 2D photocatalysts must be modified to obtain opposing charges (i.e., positive and negative charges). Notably, the zeta potential value can be used to calculate the photocatalyst’s charge. For instance, to form the 2D/2D g-C_3_N_4_/rGO by electrostatic self-assembly, the g-C_3_N_4_ was protonated by concentrated H_2_SO_4_ and HNO_3_ under mild ultrasonication to obtain the positively charged surface; the measured zeta potential value was +37.2 mV (Vinesh et al., 2020). With the aid of ultrasonication and agitation, the g-C_3_N_4_/rGO intimate interface was obtained. In addition, the stacking interactions between the sp^2^ lattices of g-C_3_N_4_ and the sp^2^ graphene lattices, as well as the hydrogen-bonding interactions between the nitrogen-containing groups in g-C_3_N_4_, are advantageous for electrostatic self-assembly. Due to the intimate interface and the introduction of rGO, the hydrogen production rate of g-C_3_N_4_/rGO (557 mol g^−1^ h^−1^) was significantly higher than that of g-C_3_N_4_ (158 mol g^−1^ h^−1^). Furthermore, the construction of the 2D/2D g-C_3_N_4_/rGO by electrostatic self-assembly facilitates the photocatalytic reduction of carbon dioxide to methane. Chemical vapor deposition (CVD) is also an efficient method for constructing 2D/2D heterostructures with intimate interfaces, such as intraplane and interplane interfaces (Zhang and Fu, 2018). Typically, in the CVD method, gas molecules are injected into a reaction chamber that has been heated to a specific temperature. For instance, the CVD-fabricated intraplane Fe_2_O_3_/g-C_3_N_4_, type-II InSe/g-C_3_N_4_ heterostructure, and g-C_3_N_4_/TiO_2_ exhibited excellent optoelectronic and photovoltaic performance (Wang et al., 2014b). Yuanzhi Hong produced Ta_2_O_5_/g-C_3_N_4_ heterojunctions using a straightforward, one-step heating procedure. Under visible-light irradiation (>420 nm) (Hong et al., 2017), the photocatalytic activity of as-prepared photocatalysts was determined by splitting water for hydrogen evolution under visible-light irradiation. Compared to pure g-C_3_N_4_, the obtained heterojunctions demonstrated significantly enhanced hydrogen production. The heterojunction of 7.5% TO/CN exhibited the highest photocatalytic hydrogen evolution efficiency, which was approximately 4.2 times that of pure g-C_3_N_4_. In addition, the 7.5% TO/CN sample exhibited excellent photochemical stability even after 20 h of photocatalytic testing. Although CVD is a powerful technique for the synthesis of 2D/2D materials, the gaseous byproducts of the process are typically extremely toxic. Moreover, using the CVD method to synthesize 2D materials on a large scale remains a formidable challenge.

### 2D layered g-C_3_N_4_-based hetero-structured photocatalysts for H_2_ production

**FIGURE 4:**
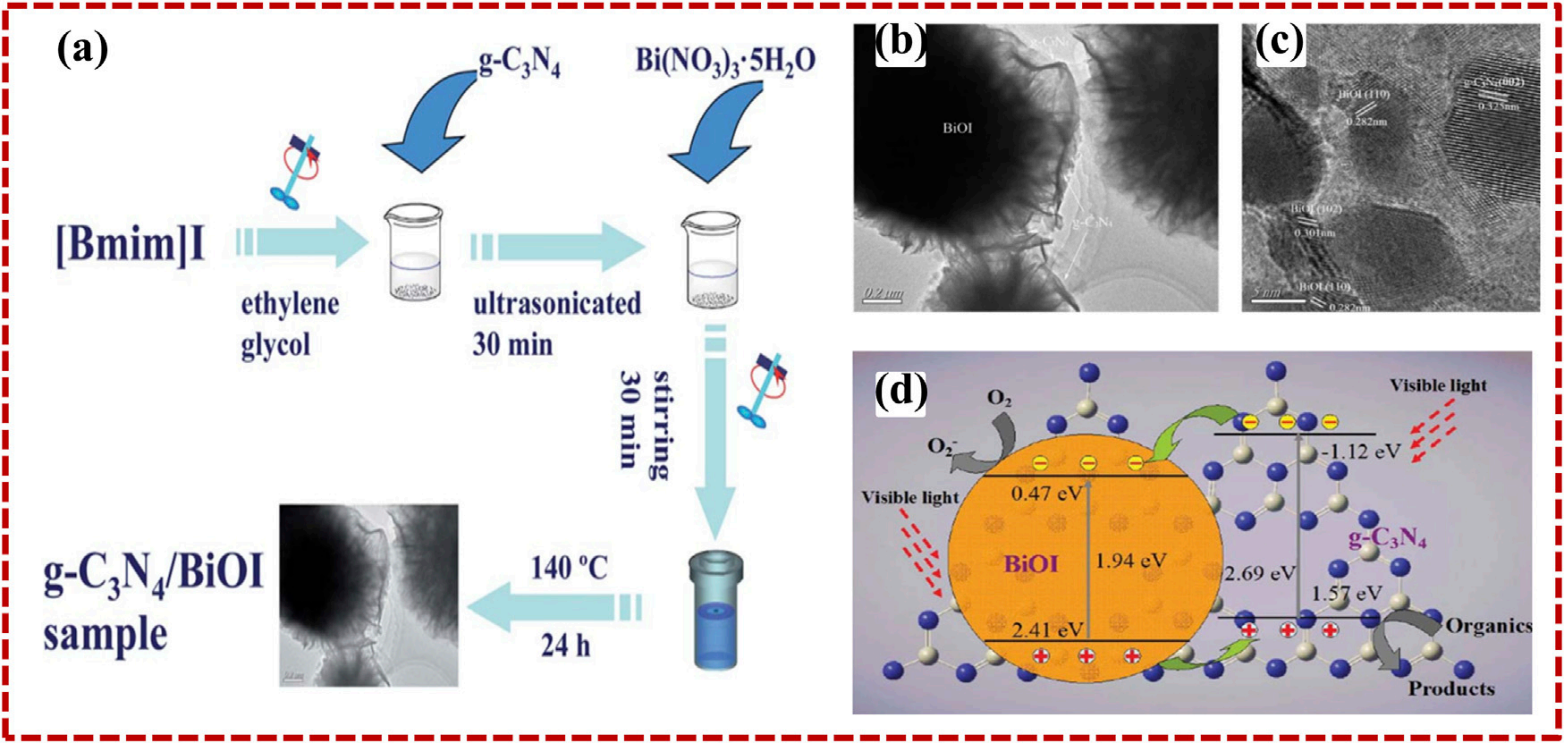
The synthesis method **(A)**, TEM **(B)**, and HRTEM **(C)** pictures of g-C_3_N_4_/BiOI. **(D)** The transferring mechanism of the photo-generated charge carriers across g-C_3_N_4_/BiOI nanocomposites. Reprinted with permission from Di et al. (2014), Copyright 2014 Royal Society of Chemistry.

As described earlier, in 2009, Wang et al. first discovered that 2D layered g-C_3_N_4_, along with featuring a 2.7 eV band gap value, is a favorable photocatalyst utilizing visible light for H_2_ production. After that, many researchers have devoted their attention to the synthesis of 2D heterostructure photocatalysts with 2D g-C_3_N_4_, as proper band construction is an important deliberation in electing the second semiconductor for engineering g-C_3_N_4_-based heterostructure photocatalysts. For example, Di et al. (2014) investigated g-C_3_N_4_/BiOI heterostructure photocatalysts through a simple hydrothermal approach; schematic representation is illustrated in Figure 4A. Transmission electron microscopy demonstrates the 2D morphology of C_3_N_4_/BiOI heterostructure (Figure 4B), and HRTEM images show the different crystal fringes patterns, which corresponds to the g-C_3_N_4_ layered structure and BiOI structure (Figure 4C). A clear interface between g-C_3_N_4_ and BiOI could be seen in Figure 4C. The intimate interface between the two constituents is helpful to transfer the charge along the interfaces. BiOI constitutes the band gap (1.94 eV), which displays a decent photo response in the visible spectrum of light (Figure 4D). Owing to the appropriate band placement of g-C_3_N_4_ and BiOI, the photo-induced hole–electron pair may be powerfully separated to operate the photocatalytic reaction.

Later on, Xu et al. (2018) employed a facile electrostatic self-assembly method to synthesize a 2D Fe_2_O_3_/g-C_3_N_4_ heterostructure photocatalyst. A robust interaction was observed among Fe_2_O_3_ and g-C_3_N_4_, originating from the transition and separation of electron and hole pair charges.

**FIGURE 5:**
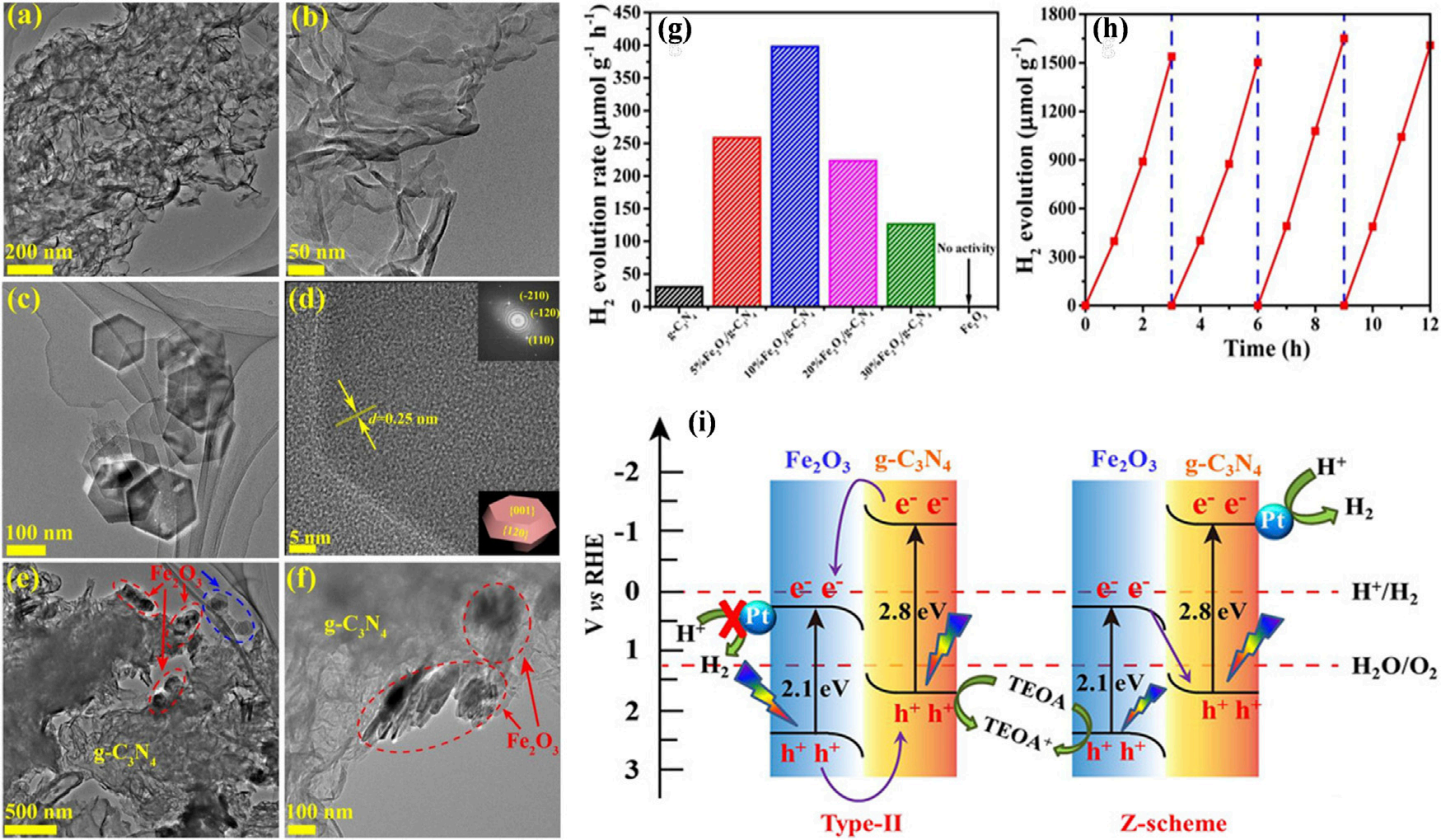
TEM photos of 2D Fe_2_O_3_/g-C_3_N_4_ **(A,B)** and 2D g-C_3_N_4_ **(C)**. HRTEM image of Fe_2_O_3_ **(D)** and the samples with 10% Fe_2_O_3_/g-C_3_N_4_ **(E,F)**. The upper right corner inset part of the image **(D)** shows Fe_2_O_3_ nanoplate FFT pattern; in contrast, the lower right corner is a depiction of Fe_2_O_3_ nanoplate facets. **(G)** Photocatalytic activities demonstration by g-C_3_N_4_ nanosheets, Fe_2_O_3_, and 2D Fe_2_O_3_/g-C_3_N_4_ heterostructure, **(H)** the stability of 10% sample Fe_2_O_3_/g-C_3_N_4_ heterostructure with visible-light of *λ* > 420 nm irradiation, **(I)** Charge transfer mechanism of traditional type-II heterojunction and direct Z-scheme. Reproduced with permission from Xu et al. (2018), Copyright 2018 Wiley Online Library.

Interestingly, the movement of charges through the 2D Fe_2_O_3_/g-C_3_N_4_ interface was found suitable for the Z-scheme. Therefore, Fe_2_O_3_/g-C_3_N_4_ photocatalysts were applied for direct photocatalytic water splitting through Z-scheme with visible-light irradiation using Pt as a co-catalyst. The morphology of the 2D Fe_2_O_3_/g-C_3_N_4_ heterostructure is depicted in Figure 5. As can be studied by the TEM in Figures 5A,B, the Fe_2_O_3_/g-C_3_N_4_ heterostructure exhibited 2D morphology. The exfoliated g-C_3_N_4_ reveals a morphology similar to curled-veil, stated as typical flexible nanosheets (Figure 5C). The FFT profile reveals the presence of six identical (1 2 0) spots, corresponding to the (0 0 1) basal plane up and the (0 0 1) basal plane down, implying that a crystal fringe distance of 0.25 nm represents (1 2 0) planes (Figure 5D). Consequently, the lower right corners of Figures 5E,F display the typical structure of a hexagonal nanoplate made of Fe_2_O_3_ with exposed facets. Based on the Fe_2_O_3_ and g-C_3_N_4_ morphologies, the TEM images with 10% Fe_2_O_3_/g-C_3_N_4_ show hexagonal nanoplate (red and blue circles in Figures 5E,F), which represents Fe_2_O_3_, while curled nanosheets were identified as g-C_3_N_4_. The nanoplates of Fe_2_O_3_ are predominantly accumulated on the edges of g-C_3_N_4_, promoting the establishment of an interface of the heterostructure. The effectiveness of the photocatalysts was assessed by their H_2_O splitting ability to produce hydrogen using visible light as an irradiation source. Triethanolamine (TEOA) was utilized to scavenge the holes. The nanoparticles of Pt played the role of co-catalysts, which were accumulated on the photocatalyst surface through *in situ* photoreduction. Figure 5G demonstrates that pristine Fe_2_O_3_ performs a very poor H_2_ generation performance, whereas g-C_3_N_4_ exhibited mild photocatalytic hydrogen generation at a 30.1 mmol h^−1^ g^−1^ rate. Interestingly, the photocatalytic performance of the Fe_2_O_3_/g-C_3_N_4_ heterostructure for the H_2_ evolution was found 398.0 mmol h^−1^ g^−1^, almost 13-times that of the pristine g-C_3_N_4_. A detailed photocatalytic hydrogen generation mechanism on Fe_2_O_3_/g-C_3_N_4_ heterostructure is illustrated in Figure 5I. As shown in Figure 5I, photocatalytic systems primarily consider two possible pathways: the conventional type-II heterojunction and the direct Z-scheme system. As seen in Figure 5I (type-II), the CB and VB energies of g-C_3_N_4_ are 1.1 and 1.7 eV, respectively. While the CB and VB values of Fe_2_O_3_ are 0.3 and 2.4 eV, to be obtained from the empirical formula (Wang et al., 2015). However, due to the low CB value of Fe_2_O_3_, electrons cannot participate thermodynamically in the photocatalytic hydrogen evolution reaction. As shown in 4i (type-II), if the composite followed the traditional type-II mechanism, g-C_3_N_4_ would transfer to the CB of Fe_2_O_3_, while photogenerated holes would transfer from the VB of Fe_2_O_3_ to the VB of g-C_3_N_4_ when exposed to visible light. In this case, the photocatalytic activity of the composite should be less than that of g-C_3_N_4_. However, the actual experimental results showed that the photocatalytic activity of the composite Fe_2_O_3_/g-C_3_N_4_ is higher than that of g-C_3_N_4_. On the basis of the preceding results and data analysis, it is proposed that a direct Z-scheme charge transfer route can occur over Fe_2_O_3_/g-C_3_N_4_ composites, thereby enhancing the photocatalytic performance in H_2_ production. In particular, when both Fe_2_O_3_ and g-C_3_N_4_ absorb photons with sufficient energy, electrons are excited from their respective VB to CB. As a result, the Fe_2_O_3_/g-C_3_N_4_ composites retain both the high oxidation ability of Fe_2_O_3_ and the high reduction ability of g-C_3_N_4_, thereby providing a substantial driving force for the water reduction reaction. The photogenerated electrons formed on the g-C_3_N_4_ surface would transfer to Pt NPs in order to participate in the surface water reduction for H_2_ evolution, whereas the photogenerated holes collected on the Fe_2_O_3_ surface could be consumed in TEOA oxidation. This direct Z-scheme charge transfer process significantly improves charge separation efficiency and provides a large driving force for the photocatalytic water reduction reaction, thereby enhancing the performance of photocatalytic water splitting.

**FIGURE 6:**
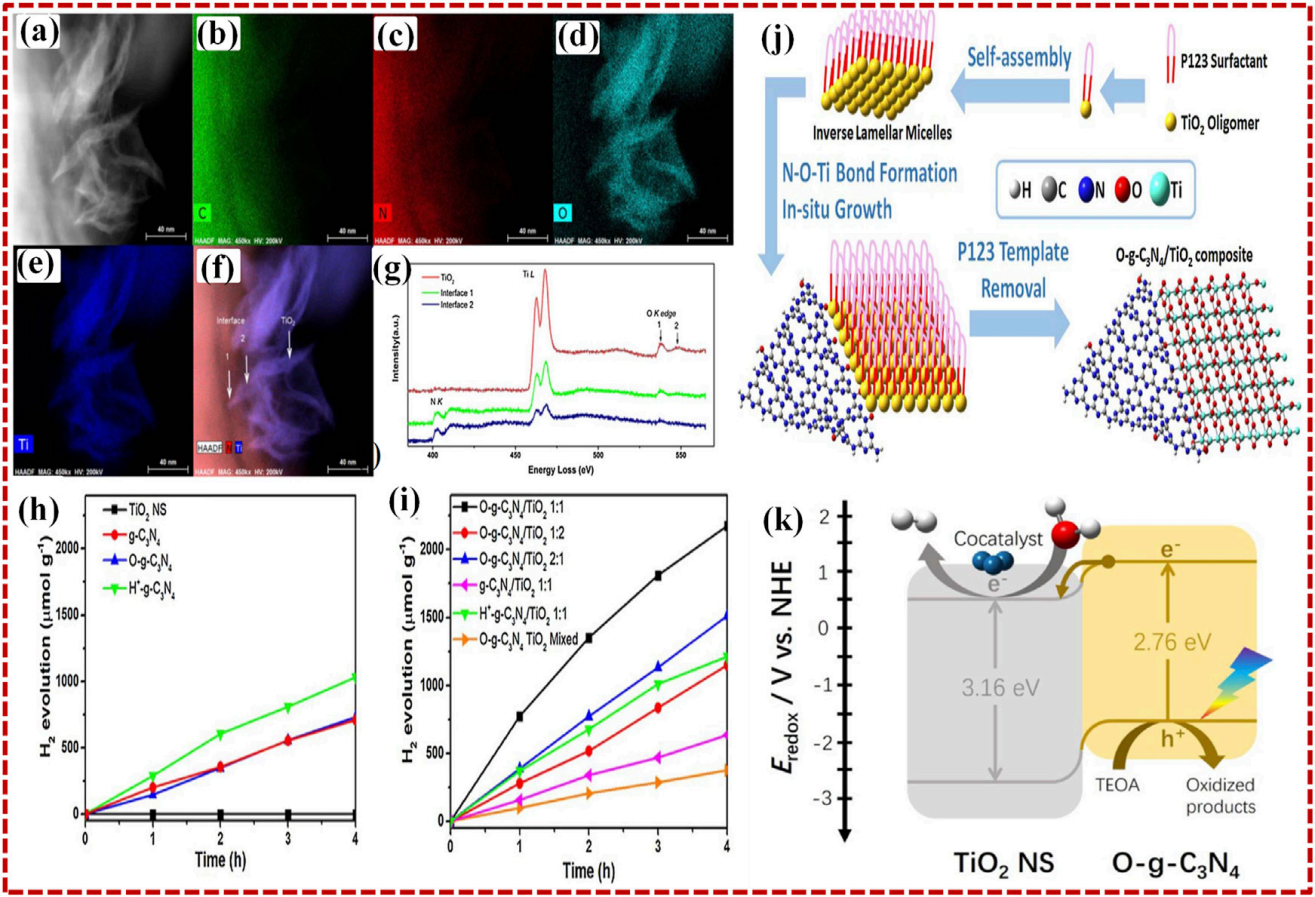
**(A)** Relevant HAADF-STEM picture and EDS elemental map of the respective regions of 2D O-g-C_3_N_4_/TiO_2_ photocatalyst C **(B)**, N **(C)**, O **(D)**, and Ti **(E)**. **(F)** superposed N, Ti, and HAADF maps revealing the interface areas and TiO_2_ leaves. **(G)** NK edge, Ti L edge, and O K edge as seen in EELS spectra of interface and TiO_2_ region. **(H)** H_2_ production plot vs. time of TiO_2_ NS, g-C_3_N_4_, O-g-C_3_N_4_, H^+^ - g-C_3_N_4_, and **(I)** composites of O-g-C_3_N_4_/TiO_2_ at ratios of 1:1, 1:2, 2:1, 2D g-C_3_N_4_/TiO_2_ (1:1), H^+^ - g-C_3_N_4_/TiO_2_ (1:1), and mixed O-g-C_3_N_4_ TiO_2_ **(J)** scheme of the fabrication of O-g-C_3_N_4_/TiO_2_ composite. **(K)** Suggested mechanism for photocatalytic H_2_ production on O-g-C_3_N_4_/TiO_2_ (1:1) composite using irradiation by visible-light. Reproduced with permission from Zhong et al. (2018), Copyright 2018 Elsevier.

Recently, Zhong et al. (2018) developed a self-assembled 2D O-g-C_3_N_4_/TiO_2_ heterostructure photocatalyst by single-pot solvothermal method for the H_2_ evolution reaction (HER) with visible light photocatalytic radiations. The two-dimensional existence of each component of the heterostructure itself gives rise to broad, unique surface areas, a marked quantum containment effect, and exposed active sites. The 2D photos of O-g-C_3_N_4_/TiO_2_ 1:1 taken by HAADF-STEM reveal a fine heterostructure formation (Figure 6A). Element mapping performed with EDX reveals that the larger nanosheet is O-g-C_3_N_4_, and the small nanosheets around its border are TiO_2_ (Figures 6B–F). In order to analyze the interface among the two elements, the electron energy loss spectra (EELS) were collected in separate contact regions 1 and 2, as well as in the virgin TiO_2_ leaf areas, as shown in Figure 6G. Figure 6H displays the photocatalytic activity for HER on the 2D O-g-C_3_N_4_/TiO_2_ with visible light irradiation. Electrons in the VB of O-g-C_3_N_4_ are excited to the CB by the incident photons with appropriate energy. TEOA trapped the photo-induced holes produced in the valence bands of O-g-C_3_N_4_, whereas photo-induced electrons passed from the heterojunction of covalent NeOeTi into the valence band of TiO_2_ nanosheet.

Photo-induced electrons eventually enter into and evenly accumulate on the surface of TiO_2_, a Pt co-catalyst, and the water splitting happens through electrons into H_2_ gas. The heterojunction identified through NeOeTi linkage caused effective charge separation at the interface and the effect of band bending, which expanded the absorption range and enhanced the photocatalytic activity of 2D O-g-C_3_N_4_/TiO_2_ heterostructure photocatalyst, as shown in Figure 6I.

**FIGURE 7:**
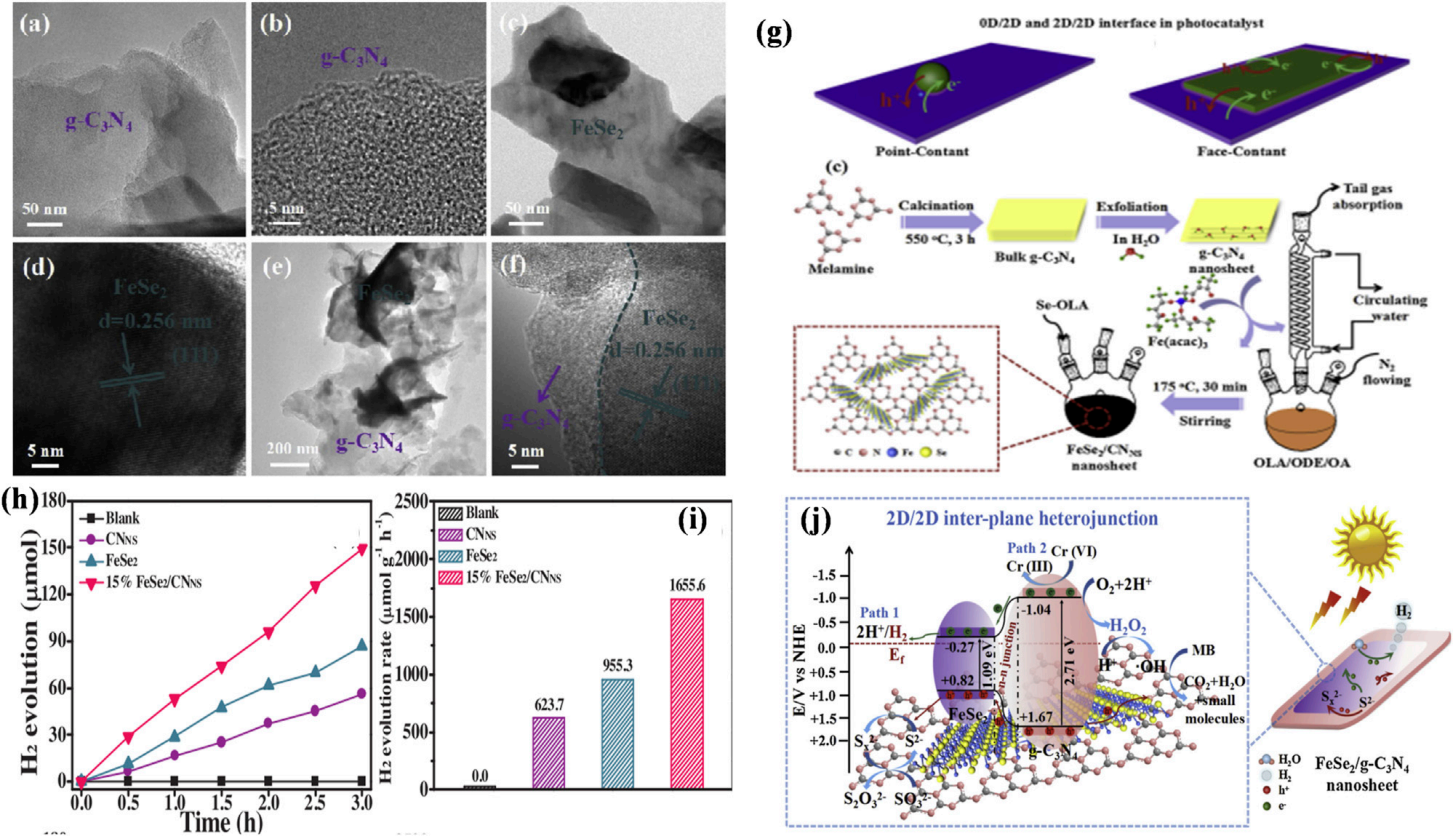
TEM image of pristine g-C_3_N_4_ **(A,B)**, FeSe_2_ nanosheets **(C,D)**, and FeSe_2_/CNNS composite of 15% sample **(E,F)**. Schematic image depicting the interfaces of **(G)** 0D/2D heterostructures, 2D/2D heterostructures and Schematic diagram for the fabrication process of inter-plane hetero-structures of 2D/2D FeSe_2_/CNNS. Photocatalytic hydrogen evolution plot **(H)** and rates **(I)** of virgin g-C_3_N_4_, FeSe_2_, and heterostructures of numerous FeSe_2_/CNNS. **(J)** Schematic depiction of the charge carrier transport in 2D/2D FeSe_2_/CNNS samples irradiated by sunlight. Reproduced with permission from Jia et al. (2020b), Copyright 2020 Elsevier.

Very recently, Jia et al. (2020b) reported that 2D 15% FeSe_2_/CNNS heterostructure exhibits superior photocatalytic hydrogen generation performance (1655.6 μmol h^−1^ g^−1^) with no co-catalyst in Na_2_S/Na_2_SO_3_ aqueous medium and excellent stability for 12 h. In addition, it also demonstrated the simultaneous elimination of chromium (VI) and methylene blue (MB) using sunlight irradiation. Most notably, relative to conventional single-step four-electron reaction, FeSe_2_/CNNS can trigger the photocatalytic H_2_O splitting to hydrogen generation through a sequential two-electron, two-step reduction reaction based on capturing of active free radical and H_2_O_2_ sensing investigation. The efficiency is simultaneously realized by such 2D/2D inter-plane hetero-structures, as seen in Figure 7.

Here, we summarize 2D g-C_3_N_4_-based heterostructure photocatalysts and compounds that contain 2D g-C_3_N_4_. Table 1 lists the experimental conditions for photocatalytic water splitting and their photocatalytic performances by using different sacrificial reagents.

**TABLE 1:** Selected reports on the 2D–2D g-C_3_N_4_-based heterostructure for photocatalytic H_2_ production.

Photocatalysts	Sacrificial agent/co-catalyst	Applications	Catalyst amount/solution composition	Quantum efficiency or hydrogen production	Light source	Ref
phosphorene/g-C_3_N_4_	lactic acid/Pt	H_2_ production	20 mg/100 ml	1.2% at 420 nm	300 W xenon lamp/(*λ* > 400 nm	Ran et al. (2018a)
g-C_3_N_4_/NiFe-LDH	CH_3_OH	H_2_ production	30 mg/30 ml	1488 mmol^2^ h^−1^	125 W pressure Hg lamp (λ > 420 nm)	Nayak et al. (2015)
α- Fe_2_O_3_/g-C_3_N_4_	/RuO_2_	H_2_ production	10 mg/100 ml	44.35% at *λ* = 420 nm	300 W xenon lamp/(*λ* > 420 nm)	She et al. (2017)
2D g-C_3_N_4_/In_2_Se_3_	TEOA	H_2_ production	20 mg/100 ml	4.8 mmol g^−1^·h^−1^	36 W LED lamp/(*λ* > 420 nm)	Zhang et al. (2020)
g-C_3_N_4_/N-La_2_Ti_2_O_7_	CH_3_OH	H_2_ production	5 mg/5 ml	10.7% at 420 nm	Asahi Spectra, mW cm−2/(*λ* > 420 nm)	Cai et al. (2017a)
WO_3_.H_2_O/g-C_3_N_4_	No sacrificial reagent	H_2_ production	100 mg/100 ml	482 μmol g^−1^ h^−1^	350 W xenon lamp/(*λ* > 420 nm)	Yang et al. (2018)
FeSe_2_/g-C_3_N_4_	Na_2_S/Na_2_SO_3_	H_2_ production	100 mg/100 ml	1655.6 μmol∙h^−1^	300 W xenon lamp/(*λ* > 420 nm	Jia et al. (2020a)
O- g-C_3_N_4_/TiO_2_	TEOA	H_2_ production	50 mg/50 ml	587.1 μmol g^−1^ h^−1^	300 W xenon lamp/(*λ* > 400 nm	Zhong et al. (2018)
g-C_3_N_4_/TiO_2_	CH_3_OH/CH_3_CH_2_OH	H_2_ production	0.15 g/100 ml	10,150 μmol h^−1^	300 W xenon lamp	Fajrina and Tahir, (2019)
Ba_5_Nb_4_O_15_/g-C_3_N_4_	oxalic acid/Pt	H_2_ production	100 mg/100 ml	2.67 mmol h^−1^ g^−1^	300 W xenon lamp/(*λ* > 400 nm	Wang et al. (2020)
ZnS/g-C_3_N_4_	Na_2_S/Na_2_SO_3_	H_2_ production	50 mg/100 ml	713.68 μmol h^−1^ g^−1^	300 W xenon lamp/(*λ* > 420 nm	Hao et al. (2018)
ZnIn_2_S_4_/g-C_3_N_4_	TEOA	H_2_ production	50 mg/60 ml	7.05% at 420 nm	300 W xenon lamp/(*λ* > 420 nm	Qin et al. (2020)
CdS/WS_2_/CN.	Lactic acid	H_2_ production	10 mg/100 ml	1174.5 mmol h^−1^ g^−1^	300 W Xe (*λ* > 420 nm)	Zou et al. (2018)
NH_2_-MIL-125(Ti)/g-C_3_N_4_	TEOA	H_2_ production	10 mg/100 ml	8.7 mmol g^−1^ h^−1^	300 W xenon lamp/(*λ* > 420 nm	Xu et al. (2017)
Nb_2_O_5_/g-C_3_N_4_	TEOA/Pt	H_2_ production	10 mg/100 ml	50.65% and 14.75% at 405 nm and 420 nm	300 W xenon lamp/(*λ* > 400 nm	Yi et al. (2021)
Mo_2_C/g-C_3_N_4_	TEOA	H_2_ production	5 mg/100 ml	6.7% at 420 nm	300 W xenon lamp/(*λ* > 420 nm	Zheng et al. (2020)
CdS/α- Fe_2_O_3_	Na_2_S/Na_2_SO_3_	H_2_ production	50 mg/100 ml	46.9% at 420 nm	300 W xenon lamp/(*λ* > 420 nm	Shen et al. (2020)
g-C_3_N_4_/Graphene/MoS_2_	TEOA	H_2_ production	50 mg/250 ml	3.4% at 420 nm	300 W Xe (*λ* > 420 nm)	Yuan et al. (2018)
Nb_3_O_7_F/g-C_3_N_4_	10% TEOA	H_2_ production	30 mg/1000 ml	1242.0 μmol h^−1^ g^−1^	300 W Xe 300 nm ≤ *λ* ≤ 1100 nm	Li et al. (2021)
Mo_2_C/g-C_3_N_4_	TEOA	H_2_ production	20 mg/90 ml	675.27 μmol h^−1^g^−1^	300 W Xe lamp, *λ* > 400 nm	Liu et al. (2022)
g-C_3_N_4_/ZnIn_2_S_4_	40 ml, 10% lactic acid	H_2_ production	8 mg/40 ml	10.92 mmol h^−1^ g^−1^	300 W Xe lamp, *λ* > 420 nm	Dang et al. (2021)
ReS_2_/CCN	10% TEOA	H_2_ production	20 mg/100 ml	3.46 mmol g^−1^ h^−1^	The xenon lamp (300 W, 250 mW cm^−2^) stimulated sunlight	Yang et al. (2022)
LaVO_4_/CN	10 ml of FFA or TEOA	H_2_ production	20 mg/100 ml	0.95 mmol g^−1^ h^−1^	The xenon lamp (300 W, 250 mW cm^−2^) stimulated sunlight	Li et al. (2022)
AgPd/2D g-C_3_N_4_	Formic acid/Sodium format	H_2_ production	100 mg/50 ml	231.6 mmol h^−1^	300 W xenon lamp/(*λ* > 400 nm)	Wan et al. (2020)

### Role of the sacrificial agent

Overall, photocatalytic H_2_ production from water using either UV-light-responsive photocatalysts or 2D g-C_3_N_4_-based visible-light-responsive photocatalysts is a low-efficiency process (Chen and Mao, 2007; Hong et al., 2013). This is primarily due to the high rates of electron and hole recombination induced by photoexcitation. To increase the efficiency of H_2_ production from water splitting, electron donors are typically required to act as a sacrificial agent, consuming holes and preventing the recombination of photoinduced electrons and holes on the semiconductor surface. Common sacrificial electron donors include Na_2_S–Na_2_SO_3_, methanol, triethanolamine, lactic acid, etc., and several studies have compared the H_2_ production rates of photocatalysts using various sacrificial agents. For instance, Hong et al. examined the H_2_ production performance of 2D g-C_3_N_4_/NiS (g-C_3_N_4_ as the photocatalyst and NiS as the co-catalyst) in solutions of triethanolamine, lactic acid, oxalic acid, and ascorbic acid. According to the results, the H_2_ evolution rate for C_3_N_4_/NiS in triethanolamine was 48.2 mol h^−1^ g^−1^, whereas the other three sacrificial agents did not support photocatalytic water splitting. These findings and other reports on photocatalysts indicate that sacrificial agents are indispensable for achieving high H_2_ evolution rates. Some studies show that sacrificial agents play a role in the dispersion of noble metal nanoparticles (co-catalysts). It has also been determined that the sacrificial agents have a significant effect on the loading amount, particle size, and distribution of various metals on the surface of g-C_3_N_4_. For instance, in methanol solution, the actual loading amount of Pt and Au is greater than in triethanolamine solution. In the presence of methanol, the distribution and size of Pt nanoparticles are improved, whereas the distribution and size of Au nanoparticles are improved in the presence of triethanolamine. As a result, the Pt- and Au-decorated g-C_3_N_4_ photocatalysts synthesized exhibit notably distinct charge transfer properties, resulting in enhanced photocatalytic activities of the same g-C_3_N_4_ photocatalyst under diverse conditions (Cao et al., 2018).

### Role of co-catalysts

Similarly, the recombination of photogenerated electron–hole pairs of g-C_3_N_4_ can be inhibited by loading the co-catalyst on the surface of g-C_3_N_4_. Briefly, co-catalysts can be categorized as singly loaded (noble-metal, metal with high abundance, and non-metallic) and co-loaded hybrid co-catalysts, which effectively promote the separation of photogenerated electron–hole and subsequently improve photocatalytic performance. Therefore, bimetallic, noble-metal co-catalysts, co-loading with different co-catalysts or one co-catalyst with various components, and the potential for solar-driven hydrogen evolution appear more promising.

Inspired by the rule of C and N in g-C_3_N_4_, also overall photocatalytic water splitting enhanced by the use of 2D g-C_3_N_4_-based hetero-structured photo-catalysts, as abundant interfaces between different components have gained attention for enhanced light absorption and facilitated photogenerated charge separation in the photo-catalysis. Recently, successful photocatalytic systems have been one of two approaches to the splitting of H_2_O into H_2_ and O_2_. A single particulate photo-catalyst is used to split water *via* one-stage excitation. A robust, reproducible particulate matter photo-catalyst that can be used under visible light satisfies various requirements. These include the best band gap and band position with enough driving potential. These materials also explain efficient charge-separation and conversion of electron–hole pairs, catalytic surface reduction, oxidation of water, and low corrosion. The preferred approach to achieving the two-stage excitation is well known as a Z-scheme process that is used to combine two photocatalytic with an electron transfer mediator.

## Conclusion

In conclusion, we have discussed 2D g-C_3_N_4_-based heterostructure photocatalysts for water splitting, specifically for H_2_ production. The 2D/2D interface plays a crucial role in photocatalytic H_2_ production for a variety of reasons. First, the integration of 2D g-C_3_N_4_ with other 2D semiconductors produces a wide intimate interface which is advantageous for separating electron and hole pairs. The construction of heterostructure junctions with band structure can be employed in facilitation to separate and transport electron and hole pairs among 2D g-C_3_N_4_ and other 2D catalysts. The widened absorption range brought on by the synergistic interaction between 2D g-C_3_N_4_ and 2D semiconductors improves the utilization of sunlight. Last but not least, the establishment of an intimate contact raises the stability of photocatalysts by reducing photo corrosion and agglomeration.

## Challenges and future perspective

Regardless of recent progress on the 2D g-C_3_N_4_-based photocatalysts, the efficacy of photocatalysts is excessively low due to the fast hole pair recombination. To overcome the current challenges, still many research efforts are needed in several aspects. First, the photocatalytic performance of 2D g-C_3_N_4_ can be enhanced by regulating the number of layers to obtain the significant yield of 2D photocatalyst. Second, the severe concern is agglomeration; when the different 2D components combine together, that would cause harm to the inimitable structural holes of the 2D morphology, which may hinder the photocatalytic performance. In this context, it is necessary to develop approaches to overwhelm the surface energies of 2D hetero-structures for enhanced stabilization of self-supporting in the 2D structural design. The third problem is the absence of research investigations on the thickness of 2D coating on the mode of action of heterostructures. Tentatively, the 2D structure’s photocatalytic efficiencies mainly depend on the thickness. In case of electrostatic self-assembly techniques, the sacrificial reagents are normally required to attain more photocatalytic activity because of the rapid recombination of the electron–hole pairs; however, it may be in contrast with their practical uses of 2D photocatalysis. Even though the building of 2D/2D boundary by linking of 2D/2D photocatalysts may assist to deviate the charge carriers to a particular level, still more effective photogenerated charge carriers associated deviations of electron–hole pairs are extremely needed. According to future aspects, multiple interfaces are needed to explore beyond the 2D/2D interfaces for the efficient improvement of photogenerated charge carriers’ segregation parallel to the interface engineering of bulk photocatalysts.
